# Greenhouse gases emission reduction for electric power generation sector by efficient dispatching of thermal plants integrated with renewable systems

**DOI:** 10.1038/s41598-022-15983-0

**Published:** 2022-07-20

**Authors:** Ijaz Ahmed, Muhammad Rehan, Abdul Basit, Keum-Shik Hong

**Affiliations:** 1 Department of Electrical Engineering, Pakistan Institute of Engineering and Applied Sciences (PIEAS), Islamabad, Pakistan; 2 School of Mechanical Engineering, Pusan National University, 2 Busandaehak-ro, Geumjeong-gu, Busan, 46241 Republic of Korea; 3 Institute for Future, School of Automation, Qingdao University, Qingdao, 266071 China

**Keywords:** Climate sciences, Environmental sciences, Energy science and technology, Engineering, Mathematics and computing

## Abstract

This research aims to contribute in developing a mathematical model for the composite probabilistic energy emissions dispatch (CPEED) with renewable energy systems, and it proposes a novel framework, based on an existing astute black widow optimization (ABWO) algorithm. Renewable energy power generation technology has contributed to pollution reduction and sustainable development. Therefore, this research aims to explore the CPEED problem in the context of renewable energy generation systems to enhance the energy and climate benefits of the power systems. Five benchmark test systems, combined with conventional thermal power plants and renewable energy sources such as wind and solar, are considered herein to obtain the optimum solution for cost and pollutant emission by using the ABWO approach. The ascendancy is not limited to environmental impacts, but it also provides the diversification of energy supply and reduction of reliance on imported fuels. As a result, the research findings contribute in lowering the cost of fuel and pollutant emissions, correlated with electricity generation systems, while increasing the renewable energy usage and penetration. Finally, the performance and efficacy of the designed scheme have been fully validated by comprehensive experimental results and statistical analyses.

## Introduction

The growing demand of electricity and power generation from fuel contribute significantly to greenhouse gases emissions and global climate change^1,2^. This detrimental role is becoming more pronounced as the economic and industrial advancements are accelerating throughout the world. To this end, there has been a tendency for emissions reduction in the energy industry via the integration of renewable energy sources (RES) such as wind and solar alongside the traditional thermal plants. Energy emission load dispatch (EELD) is a critical problem for optimizing the power systems energy along with climate advantages under various equality and inequality constraints^3,4^. Economic load dispatch (ELD) is currently classified into static ELD and dynamic energy emission dispatch (DEED); the later one takes both cost and emission objectives into account. Additionally, there is another high-performance composite energy emission dispatch (CPEED) problem, which integrates the renewable energy production into DEED. CPEED considers both renewable sources of energy (such as wind or solar) and the multi-objective economic emission function of the conventional thermal power plants. Further, complex constraints associated with the CPEED optimization problem are more difficult to handle due to their complex and multi-dimensional nature. When addressing the CPEED issue, it is vital to consider the limits imposed by thermal power plants such as network load demand constraint, generator operating capacity limits, ramp-rate limit of generating units, and forbidden operating regions (FOR) in addition to those imposed by renewable energy sources. As a result, CPEED is a highly difficult optimization problem with multiple objectives. Many experts are currently engaged in exploring the CPEED problem exclusively through the lens of wind and photo voltaic generation^5,6^.

To investigate the effect of renewable energy production on power systems, the research in^7^ considers both wind and photo-voltaic renewable energy production in the CPEED objective function. Further, implementation of a novel solution, based on state of the art ABWO computing approach, has been investigated for an optimum solution of fuel cost and air emissions by considering RES. The numerical method and artificial intelligence algorithm are the two types of techniques that have been developed so far to solve EELD and DEED nonlinear objective functions, respectively. Numerical methods are generally iterative techniques, such as gradient iterative optimization algorithm^8^, lambda incremental cost iteration approach^9^, linear programming^10^, quadratic programming^11^ and Newton-Raphson schema^12^ to solve convex energy load dispatch (CELD) problems. Various artificial intelligence and evolutionary programming techniques such as particle swarm optimization^13^, enhanced sailfish approach (ESFA)^14^, genetic algorithm^15,16^, real-coded chemical reaction optimization^17^, bio-geography-based optimization^18^ and hybrid stochastic optimization (HSO)^19,20^ have been developed to resolve complex-constrained multi-objective real-world engineering problems.

The EELD issue has been extensively researched throughout the world, and numerous methods for investigating EELD problem have been proposed in the recent literature. Qiao et al.^21^ proposed a novel integrated system for electric car and wind power that combines charging and discharging of electric cars in order to mitigate the renewable power penalty costs involved and to deal with underestimated wind energy accessibility. To demonstrate the implemented model’s feasibility and efficiency, the authors proposed a bi-objective differential evolution technique that uses non-dominant sorting. However, the study has some deficiencies because the optimization strategy’s generalisation and convergence stability are needed to be improved. Moreover, CPEED problem ignores the effects of wind power variability on energy costs. The authors in^22^ suggested exponential moving average (EMA) scheme to resolve the wind energy emissions dispatch problem. The EMA algorithm works well for nonlinear problems with many variables. The study ignores systems with high wind turbine penetration, assuming idealistic wind power curves. Chen et al.^23^ proposed a conditional value credibility (CVaC) model for hedging multi-period energy emissions dispatch, integrated with random fuzzy wind power.

The largest portion of the world’s energy needs is met by carbon and hydrogen-based fuels, which are in limited supply^24^. These pollution sources cause global warming, extreme weather, and the ozone hole breach^25^. In contrast, renewable systems with abundant availability and zero carbon footprint are ideal for the aforementioned issues. However, they have their problems, including low energy density, instability, and unpredictability^26^. In order to develop a robust, sustainable, and cost-effective energy system, the integration of diverse energy sources into an electric power grid has shown to have a contribution to address fossil fuel and RES-related concerns^27^. Renewable energy is unpredictable and often unavailable. Wind and solar energies are affected by changing weather conditions. RES sources are also impacted by the climate change, and the modification of climatic influences can have a variety of consequences on different types of RES. For example, there is a chance that biomass crop yields can fluctuate in water streams, which will ultimately have severe consequences for hydroelectric plants. The increase in temperature reduces the photovoltaic energy system’s output. In addition, a spike in temperature can alter wind speed, currents, and patterns, ultimately affecting the production of wind power plants^28^. The bi-objective cross-entropy approach was developed in^29^ to tackle the uncertainty about intermittent power sources and practical constraints for various renewable energy systems, such as wind, hydro, thermal, and photo-voltaic systems. The same model with the intermittent nature of wind energy is solved by a chaotic sine-cosine algorithm in^30^. Due to the increasing penetration of renewable energy, a modified multi-objective cross entropy algorithm is proposed in^31^ to solve the EELD problem with uncertain RES. On the basis of the DEED problem, the CPEED problem considers the impact of renewable energy production on fuel costs and greenhouse gases emission. However, the associated convex and non-convex constraints of the CPEED problem are much more complicated than those of the static ELD and EELD problems, necessitating the use of algorithms with significantly greater solving potentials. Wu et al. in^32^ proposed a grid-connected integrated energy system for the synergistic interactions between electric powers, temperature, and gas energy flows, in which the flange between bio-fuels, solar, and wind energy is properly explored. Further, anaerobic digestion heating is used to maintain a proper heat for anaerobic digestion bio-gas. A new bi-objective virus colony optimizer (VCO) method was proposed, based on anti-filtering theory and fuzzy strategic planning, by Zou et al.^33^ for finding the best possible solution between the Pareto sets. At various points throughout the working day, wind power generators and plug-in electric mobility (PEM) are being integrated into the bi-objective DEED problem, which seeks to minimize the cost of wind-thermal electrical energy while also minimizing the emissions produced by fossil-fueled power plants.

There has been a considerable increase in the green energy integration with the conventional energy systems around the world particularly in western countries. This has brought new operational issues such uncertain behavior of climate conditions. A lot of theoretical and empirical investigations have been conducted on the uncertainty modelling for RES. Monte Carlo Simulation (MCS) is widely-applied as a stochastic method that offers high precision. Researchers in^34^ proposed a modified Metropolis-coupled Markov chain MCS scheme to anticipate the stochastic behaviour of numerous unpredictable elements in the planning of RES integrated energy hubs. Solar irradiance, wind speed, and energy tariff are considered as uncertain parameters in the objective function. System dynamics and MCS are integrated into a time frame technology value approach for RES in the work of Jeon et al.^35^. The framework of these complicated interactions can be effectively modelled using system dynamics. Numerous researchers have applied the MCS approach for RES uncertainty such as probabilistic power flow analysis^36^, optimal sizing of power grids^37^, short-term RES production uncertainty^38^, and RES power production risk analysis^39^. Alternatively, probabilistic analytical strategies can model the unknown RES component for accurate and reliable results. The work of Zubo et al.^40^ has devised a convolution approach to model the hybrid power system for optimal sizing and placement of distributed RES generator uncertainties in power networks. Meng et al.^41^ suggested an optimization method, featuring the time-based Taylor series, for modeling the uncertain parameters in structural designs. Several other studies such as point estimation for hybrid power networks^42^, bi-stage approach^43^ and decentralized cooperative techniques^44^ have been used to model the RES uncertain behavior in energy grids. 

To bolster the differential evolutionary algorithm’s adaptive capability, the author in^45^ proposed a self-adaptive variable search approach as well as a local search operator, for solving the EELD problem in high dimensions, containing highly nonlinear terms, non-smoothness, and non-convexity. However, the work ignores the fact that such algorithms are prone to local values and pushed to their limits, limiting their utility in solving the CPEED problem. In the light of the growing share of modern power generation systems, it is necessary to investigate the impact of photo-voltaic and wind power production on the energy and environmental benefits of power systems. The conventional methods like^46–50^ have several research gaps as these methods have not accounted a comprehensive, generic, and highly conflicting optimization problem of CPEED by incorporating wind and solar generation uncertainties, load demand limits, generating capacity limitations, and ramp-rate impacts of generation machines. In addition, these methods can also be improved for better cost value and for better convergence time. 

To bridge the above-mentioned research gaps in the literature, we redesign the EELD objective model by taking into account both wind power generation and photo voltaic power generation as RES, in addition to the conflicting relationship between economic and environmental benefits over time. Integration of RES into conventional energy hubs will be beneficial in numerous ways. First, it contributes to the reduction of greenhouse gases emission in power hubs in order to maintain the environment. Consideration of international emissions control agendas, such as the United Nations sustainable development goals, will be facilitated by the scheme. In addition, it will help states to deal with geopolitical concerns by lowering their dependence on imported fuels. There are two main purposes of the present study: First, the CPEED must be solved, and a competitive solution must be found to reduce power generation costs and pollutant emissions to the lowest possible levels. Second, effective solutions of CPEED must meet the non-convex, high dimensional constraints of EELD such as FOR, VLE, and power output balancing limitations with a part of renewable energy probabilistic model, featuring the combined influencing uncertainty parameter of wind and solar profiles. Our work’s primary philanthropy is as follows:

1. A practical and more generic multi-objective model of CPEED is considered herein for energy hubs, integrated with RES. The presented model considers most of the real-world complex system-level constraints such as capacity constraint, ramp-rate limitation, load balancing limitation, VLE limitation of thermal plants and RES uncertainties for both solar and wind as compared to^46–49^.
2. The existing ABWO approach is applied to solve the difficult probabilistic multi-objective CPEED model with multi-dimensional restrictions associated with traditional thermal plants and integrated RES. Owing its feature of elegance and adaptability, ABWO results in effective fuel cost and pollutant emissions reductions for the considered five test systems, ranging from small-to-large scales. It is clear from the results that ABWO outperforms other approaches as compared to^51–56^.
3. The proposed techniques provide better results in terms of early convergence in the presence of non-convex, non-linear, and high-dimensional constraints due to the cannibalism mechanism in ABWO, and they offer higher optimization performance and lower computational cost. Compared to current state-of-the-art algorithms, the proposed algorithm has better convergence and execution time. The mutation step validates the equilibrium between the exploitation and exploration phases. Therefore, the proposed approach is more effective at avoiding the local solutions within a given search space as compared to^51–57^.

### Problem formulation and CPEED modeling

A framework for CPEED can reduce the cost of generation as well as the pollutant air emissions by scheduling the optimum generation from generating resources. There is no universal solution to obtain a single feasible optimized solution set for both models, simultaneously. These two conflicting issues yield various solution sets of Pareto optimal allocation, and choice goes to the decision-maker to attain a solution according to the priorities. The objective function of CPEED can be expressed as in Eq. (1)^58^.

1 $$\begin{aligned} Min\left( F \right) = \left\{ {{F_{TC}},{E_{TE}}} \right\} . \end{aligned}$$

where *F* denotes the multi-objective problem to be minimized and $F_{TC}$ and $E_{TE}$ are the total fuel cost and total emissions, respectively.

### Fuel cost modeling

The model for economic generation cost can be expressed by quadratic function and is shown in Eq. (2)^59^.

2 $$\begin{aligned} {f_{EGC}} =\sum \limits _{i = 1}^N {{f_i}({p_i})} = \sum \limits _{i = 1}^N {{u_i}p_i^2 + {v_i}{p_i} + w_i}, \end{aligned}$$

where $u_i,v_i$, and $w_i$ are the fuel cost coefficients of particular thermal power plant. $f_{i}$ denotes the thermal generation cost, and $p_i$ is the generated power of *N* committed units.

### Fuel cost modeling with VLE

VLE introduces the rippling effect on the cost curve of thermal power plants and makes the function as highly non-convex. The modelling of fuel cost with VLE can be shown in Eq. (3)^60^.

3 $$\begin{aligned} {f_{EGC}}= \sum \limits _{i = 1}^N {{f_i}({p_i})} = \sum \limits _{i = 1}^N {{u_i}p_i^2 + {v_i}{p_i} + w} + \left| {{e_i}\sin \{ {f_i} \times (p_i^{\min } - {p_i})\} } \right| , \end{aligned}$$

where $\left| {{e_i}\sin \{ {f_i} \times (p_i^{\min } - {p_i})\} } \right|$ is the VLE on the cost curve of $i^{th}$ unit.

### Cost modeling of solar generation

The mathematical model for solar generation cost can be expressed as in Eq. (4)^51^.

4 $$\begin{aligned} {f_{SGC}} = \sum \limits _{k = 1}^{{N_s}} {{S_{p,k}} \times {b_i}{g_k}}, \end{aligned}$$

where $f_{SGC}$ represents the solar generation cost. $N_s$ and ${S_{p,k}}$ are the number of solar panels and power in megawatts, respectively.

### Cost modeling of wind generation

The mathematical model for wind generation cost has the form (5)^51^.

5 $$\begin{aligned} {f_{WGC}} = \sum \limits _{j = 1}^{{N_z}} {{W_{p,j}} \times {C_{aj}}}, \end{aligned}$$

where $f_{WGC}$ represents the wind generation cost. $N_z$ and $W_p$ are the number of wind plants and power in megawatts, respectively.

### Total cost model with VLE

The mathematical model for total cost with VLE can be expressed as in Eq. (6)^61^.

6 $$\begin{aligned} F_{TC}=\sum \limits _{i = 1}^N {{u_i}p_i^2 + {v_i}{p_i} + w} + \left| {{e_i}\sin \{ {f_i} \times (p_i^{\min } - {p_i})\} } \right| +\sum \limits _{k = 1}^{{N_s}} {{S_{p,k}} \times {b_i}{g_k}}+\sum \limits _{j = 1}^{{N_z}} {{W_{p,j}} \times {C_{aj}}}, \end{aligned}$$

where $F_{TC}$ is the total cost as shown in Eq. (1) and the model consists of sum of all associated costs of load dispatch with VLE.

### Total greenhouse gasses emission model

In this study, a comprehensive model of pollutant emissions is used to simulate the relationship between pollutant emissions and thermal power unit output, which is a quadratic function along with an exponential function. Usually, thermal power plants burn different fossil fuels to generate power, while the wind and solar energy systems are considered as clean sources because they do not emit pollutant. The emissions objective function does not need to consider the emissions characteristics of wind and photo voltaic power generation, and it can be written as mentioned in Eq. (7)^5^.

7 $$\begin{aligned} E_{TE}=\sum \limits _{i = 1}^N {({X_i}} p_i^2 + {Y_i}{p_i} + {Z_i} + {\theta _i}\exp ({\sigma _i}{p_i})), \end{aligned}$$

where $X_i$, $Y_i$, $Z_i$, $\theta _i$ and $\sigma _i$ are the emissions coefficients of all committed generator. Scientists have focused on two aspects of solving multi-objective problems commonly known as the priori and posterior approaches. The priori approaches reduce a multi-objective problem to a single problem by applying appropriate weights to achieve a feasible compromised solution that meets the requirements. The mathematical modelling is shown in Eq. (8)^61^.

8 $$\begin{aligned} min \left( F \right) = h \times {F_{TC}} + {E_{TE}}\left( {1 - h} \right) . \end{aligned}$$

CPEED is a complex engineering problem, and it consists of different nonlinear system limitations. To obtain the optimal set of solutions, the proposed ABWO strategy will satisfy all CPEED related constraints. The combined power generated from all sources, that is, thermal wind and solar must meet the load demand and transmission loss, as depicted in Eq. (9)^51^.

9 $$\begin{aligned} \sum \limits _{i = 1}^{{N^{thermal}}} {\sum \limits _{i = 1}^{{N^{solar}}} {\sum \limits _{i = 1}^{{N^{wind}}} {\left( {{p_i}} \right) } = } } p_i^{Tx.Loss} + p_i^{load}, \end{aligned}$$

where $N^{thermal}$, $N^{wind}$, and $N^{solar}$ denote the number of thermal, wind and solar committed units. $p_i$ is the generated power, $p_i^{Tx.Loss}$ and $p_i^{load}$ are the transmission losses and load power demand, respectively. Furthermore, the transmission loss of utility network can be modeled as in Eq. (10).

10 $$\begin{aligned} p_i^{Tx.Loss} = \sum \limits _{i = 1}^N {\sum \limits _{j = 1}^N {{p_i}{\beta _{ij}}{p_j} + \sum \limits _{i = 1}^N {{\beta _{oi}}{p_i} + {\beta _{oo}}} } }, \end{aligned}$$

where $\beta _{ij}$, $\beta _{oi}$ and $\beta _{oo}$ are loss coefficients matrix^62^.

### Machine limitation of generating units

The machines limitation constraints that can be modeled as in Eq. (11)^63^.

11 $$\begin{aligned} p_i^{\min } \le {p_i} \le p_i^{\max }, \end{aligned}$$

where $p_i^{\min }$ and $p_i^{\max }$ are the minimum and maximum generated powers, machine can deliver.

### Ramp-rate limitation

The ramp-rate limits can be modelled for generated $p_i$ by a generator as given in Eq. (12)^59^.

12 $$\begin{aligned} \max \left\{ {p_i^{\min },p_i^o - D{R_i}} \right\} \le {p_i} \le \min \left\{ {p_i^{\max },p_i^o + U{R_i}} \right\} , \end{aligned}$$

where $U{R_i}$ and $D{R_i}$ are the upper and down ramp-rate limitations. Since some generating units have physical limitations, their actions are limited to specific operating regions, likely to result in input-output characteristics that are discontinuous in nature. The modeling for such type of regions in input-output characteristics is given in Eq. (16)^64^.

13 $$\begin{aligned} \{p_i^{\min } \le {p_i} \le p_{i,1}^L\},\{p_{i,H - 1}^U \le {p_i} \le p_{i,H}^L\},\{p_{i,nz}^U \le {p_i} \le p_i^{\max }\}, where H = 2, \ldots , nz. \end{aligned}$$

### Wind probability density function

Wind energy generation is heavily reliant on wind speed variability. Numerous techniques have been used to portray the randomness of wind speed characteristics. The Weibull probability density function (WPDF) is used to model the wind speed attributes, and it is used to define the stochastic feature of wind speed profiles. The mathematical expression of WPDF is as shown in Eq. (14)^65^.

14 $$\begin{aligned} WPDF\left( {{V_{CWD}}} \right) = \left\{ {\frac{\Pi }{\mathrm{T}}\left( {\frac{V}{\mathrm{T}}} \right) ^{\Pi -1} \cdot \exp \left( { - {{\left( {\frac{V}{\mathrm{T}}} \right) }^\Pi }} \right) V > 0,} \right. \end{aligned}$$

where *T* and $\Pi$ represent the scale and shape parameter, respectively. $V_{CWD}$ shows the current speed of wind in (*m*/*s*). The power output of wind is computed using the speed-power curve via Eq. (15).

15 $$\begin{aligned} {W_p} = \left\{ \begin{array}{l} 0, \quad \left( {V < {V_{in}}ORV \ge {V_{out}}} \right) \\ \frac{{{w_r} \quad \left( {V - {V_{in}}} \right) }}{{{V_r} - {V_{in}}}}\left( {{V_{in}} \le V \le {V_r}} \right) \\ {w_r} \quad \left( {{V_r} \le V \le {V_{out}}} \right) \end{array} \right. \end{aligned}$$

where ${w_r}$ represents the rated wind turbine power and $W_p$ is the generated output power. $V_r$, $V_{in}$ and *V* show the cut in/off and rated speed respectively.

### Solar probability density function

Unlike the wind energy, the electricity production of the photo-voltaic module is heavily reliant on solar radiations, environmental temperature and module performance characteristics. In this work, we apply beta distribution (BPDF) to represent percentages or proportions of outcomes. The mathematical modelling of solar energy using BPDF is depicted in Eq. (16)^66^.

16 $$\begin{aligned} {f_{\left( {BPDF} \right) }}\left( \Phi \right) = \left\{ \begin{array}{l} \frac{{\Gamma \left( {R + TE} \right) }}{{\Gamma \left( R \right) \Gamma \left( {TE} \right) }} \times {\Phi ^{R - 1}}{\left( {1 - \Phi } \right) ^{TE - 1}}\\ for \quad 0 \le \Phi \le 1, \quad R \ge 0,TE \ge 0\\ 0,otherwise \end{array} \right. \end{aligned}$$

Here *R* and *TE* represent the ${f_{BPDF}}$ parameters, $\Phi$ is the electromagnetic radiation power per area, and $\Gamma$ is gamma function of BPDF. By using mean $(\mu )$ and standard deviation $(\varepsilon )$, the function can be written as follows:

17 $$\begin{aligned} R = \mu \left( {\frac{{\left( {\mu \left( {\mu + 1} \right) } \right) }}{{{\varepsilon ^2}}} - 1} \right) , \end{aligned}$$

18 $$\begin{aligned} TE = \left( {1 - \mu } \right) \left( {\frac{{\mu \left( {\mu + 1} \right) }}{{{\varepsilon ^2}}} - 1} \right) . \end{aligned}$$

As stated earlier, the dependency for power generation in photo-voltaic modules relies on the solar irradiance and the temperature of cells. It can be formulated via Eq. (19).

19 $$\begin{aligned} {S_p}(t) = {N_{series}} \times {N_{Parallel}}[ S_{p}(stcon) \times \frac{S(t)_{rad}}{S_{rad.stcon}} \times [ 1- \chi \times (U_{cell} - {U_{cell.stcon}})]]. \end{aligned}$$

The temperature of cells can be determined from Eq. (20), where ${S_p}(t)$ denotes the solar radiation incident on the photo-voltaic module at time interval *t*. $S_{p(stcon)}$ and $S_{rad.stcon}$ represent the solar power and radiance in the standard test conditions. $\chi$ is the coefficient of temperature while $U_{cell}$ and $U_{cell.stcon}$ are the cell temperature and temperature at standard conditions. $U_{ambient}$ and $U_{normal.temp}$ are the environmental and normal temperature, respectively.

20 $$\begin{aligned} {U_{cell}} = {U_{ambient}} + \frac{{S{{(t)}_{rad}}}}{{{S_{rad.stcon}}}} \times ({U_{normal.temp}} - 20). \end{aligned}$$

Traditional thermal power plants can provide electricity regardless of the weather, but RES rely on the climatic strength to generate power, making it imperative to have an accurate and reliable model to deal with the unpredictability of climate circumstances. In this study, we used beta-distribution function (16) and Weibull probability density function (14) to model the uncertainty in RES. The wind PDF is computed using (14), where *T* is the scaling factor and $\Pi$ is the wind turbine blade profile shape factor. The values of *T* and *V* for the Kings park site are 3.23 and 5.8 metres per second, respectively. Once the unpredictability of the wind has been classified as a stochastic process, the output power of the wind generator can be measured as a random variable by transforming wind speed into output power. Equation (15) can be used to determine the output power of the wind based on the wind velocity $W_P$, and can used in wind cost function Eq. (5) to compute the cost. To account for the uncertainty in the solar cost function, solar irradiance is classified as a random variable using the beta distribution. ${f_{\left( {BPDF} \right) }}\left( \Phi \right)$ is the distribution function and is characterized as a random variable of solar irradiance (kw/$m^2$). Now, in order to acquire the power that the solar panel produces, we have used $S_{P,k}=S_{P0}\times {f_{\left( {BPDF} \right) }}\left( \Phi \right)$, which further is used in the solar cost function equation (4) to compute the cost. It is pertinent to mention here that we have formulated a complex and more practical model of the economic emission dispatch which includes practical contagious system-level limitations for example, VPLE, ramp-rate limitations and system losses as compared to the similar ABWO framework^50^. Additionally, we have also integrated the renewable energy resources in our work in the form of wind and solar sources in comparison to the mentioned research study.

## Proposed computing paradigm

**Figure 1:**
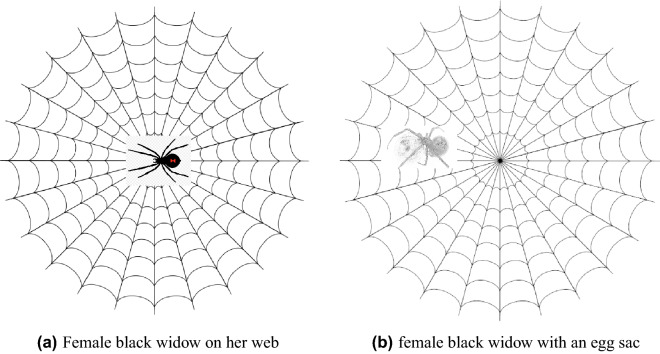
Female black widow representation for web and egg sac.

This section discusses the details on parameters and implementation of ABWO to resolve the CPEED problem. Hybrid power systems play a critical role in reducing reliance on imported fossil fuels and lowering atmospheric contamination. The penetration of RES in traditional energy sources introduce many challenges in power system operations. To handle the complex multi-dimensional constraint for RES integration modelling with thermal plants, we need a powerful computing framework that attains a feasible solution set by satisfying all associated constraints. Hayyolalam and Pourhaji Kazem^67^ proposed a new evolutionary optimization algorithm, based on the mating behaviour of black widow spiders. It has been used to resolve a variety of scientific and engineering problems due to its ease of use, flexibility, and speed. A black widow feminine stays out of sight during the sunlight period and spins her web during the night. The widow usually lives along the same location for much of her adult life. When a female black widow is ready to mate, she sprays pheromone on specific areas of her net in order to attract the attention of the male. By reducing the attractiveness of females webs to rivals, the first male to enter the web makes females webs less appealing to them. The female eats the male during or after mating, and then she transfers the eggs to her egg sac, which she then consumes^68^. Upon hatching, the young cannibalise each other, a short time later, they leave their mothers’ web and may even eat her. Only the fit and strong individuals will survive^69^. This is the global optimum of the objective function, and it is the best one. Figure 1a depicts a female black widow on her web, while Figure 1b shows a female black widow with an egg sac on her web.

In the ABWO algorithm, each spider represents a potential solution. They try to reproduce a new generation of spiders in pairs. After or during mating, female black widows eat the male black widows. Then she releases the sperms she had stored in her sperm theca into the egg sacs. After 11 days, spider lings emerge from the egg sacs and sibling cannibalism is discerned during their stay on the mother’s web for a few days to a week.

### Logical steps of ABWO computing paradigm

To solve CPEED optimization problem, the values of problem variables must form a suitable structure. This structure is known as a chromosome in genetic algorithms and particle position in particle swarm optimization, but it is known as a widow in ABWO. Each CPEED problem in ABWO has a potential solution as a black widow spider. Each black widow spider represents a problem variable. In this paper, the structure is treated as an array to solve benchmark functions. A $N_{var}$ dimensional optimization problem’s solution is represented by the $1\times N_{var}$ array. The fitness of a widow is calculated by the fitness function. The logical steps of ABWO are as follow.

#### Initial population

The candidate ${N_{pop}}\times {N_{var}}$ size widow matrix is generated with an initial population of spiders to initiate the optimization algorithm. The parents’ pairs are then randomly chosen to produce a new generation by mating the black male widow that the female eats during or after that time.

#### Procreate

Because the pairs are no longer dependent on one another, they begin to mate in order to produce a new generation of living creatures. A random selection of parents is made during the procreation process. Brand new generations (spider babies) are produced according to the following mathematical expression.

21 $$\begin{aligned} \left\{ \begin{array}{l} {Q_{spider.baby(1)}} = {D_{rv}} \times {O_{prnt(1)}} + (1 - {D_{rv}}) + {O_{prnt(2),}}\\ {Q_{spider.baby(2)}} = {D_{rv}} \times {O_{prnt(2)}} + (1 - {D_{rv}}) + {O_{prnt(1),}} \end{array} \right. \end{aligned}$$

where $O_{prnt(1)}$ and $O_{prnt(2)}$ represent the parents and ${Q_{spider.baby(1)}}$ and ${Q_{spider.baby(2)}}$ denote spider babies or new generations, and $D_{rv}$ is a random vector between 0 and 1.

#### Cannibalism

Population decimation happens in three distinct ways during the cannibalism phase. The first type of population destruction occurs when a female spider consumes a male spider. In the second version, larger spiders consume smaller spiders. In the third category, infants consume their mothers. The cannibalism rating (CR) in ABWO is used to calculate the population’s rate of survival^70^.

#### Mutation

A mutation function is applied to a random subset of population members during the mutation phase. Each of the selected solutions swaps two components in the array at random. The mutation rate is used to calculate mutepop^71^.

### Convergence of ABWO

It is possible to consider three stopping conditions for ABWO algorithms, similar to other evolutionary algorithms, which are as follow.

1. The number of iterations can be predetermined.
2. Observation of the best widow’s fitness value remains the same after several iterations.
3. The desired level of precision is achieved.

### Parameter setting

Identifying parameters is an important step in the proposed algorithm. It’s the cannibalism, procreation, and mutation rates setting. Table 1 lists the chosen values for the parameters in this paper for ABWO tuning.

**Table 1:** Parameter setting of ABWO.

Input setting of ABWO	
Input parameter	Parametric value
Percent of crossover	0.60
Percent of mutation	0.60
Percent of cannibalism	0.44

### Implementation of ABWO for CPEED

**Figure 2:**
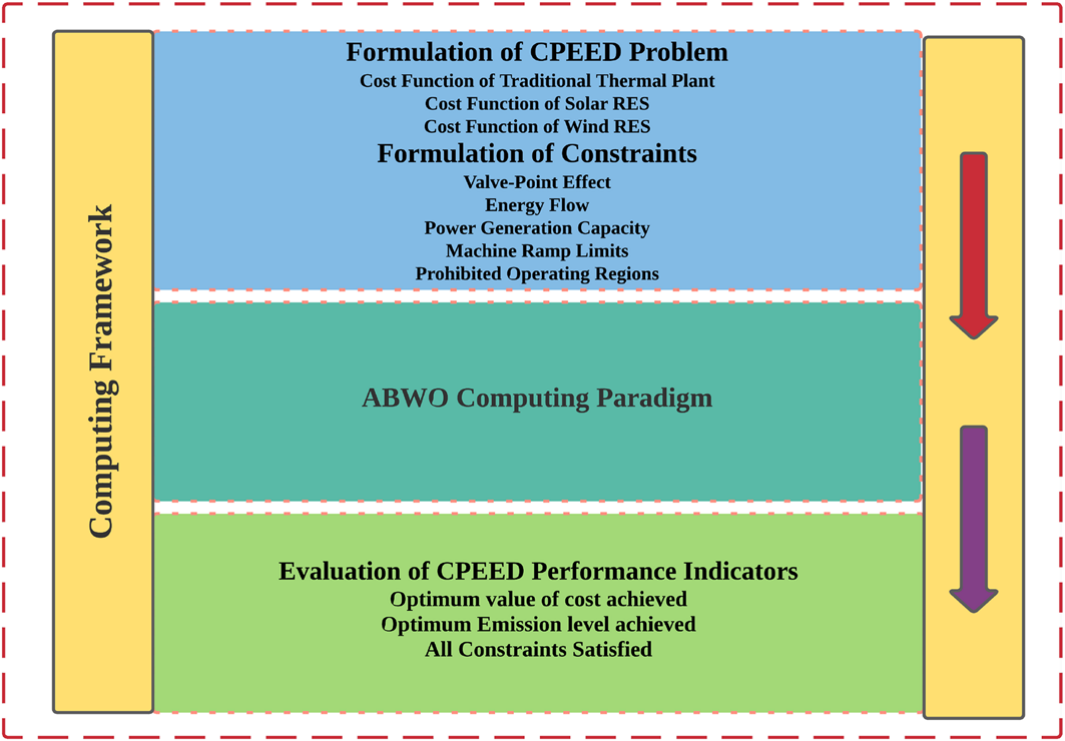
Computing framework of proposed CPEED.

**Figure 3:**
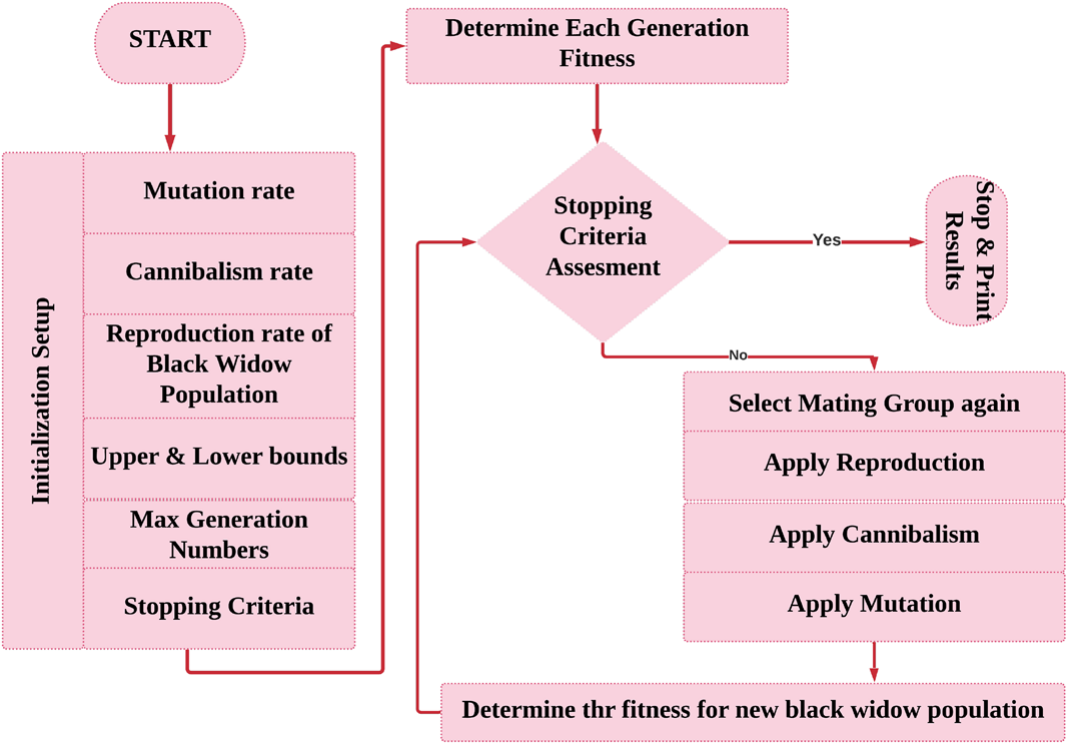
ABWO flowchart.

The computing framework for implementing ABWO for CPEED is depicted in Fig. 2, which includes objective functions, modelling equations, and an ABWO approach with performance criteria. The flowchart in Fig. 3 illustrates the problem initialization parameters and CPEED fitness stopping criterion. Algorithm 1 states the complete details for instituting ABWO for CPEED. Figure 2 depicts the working strategy of ABWO computing paradigm. All CPEED cost functions are converted into a single objective function according to the parametric setting in Table 1 and fed into the optimizer framework. The framework starts optimizing the objective function according to the flow chart in Fig. 3 and attains the best optimist set of values by satisfying all constraints in consonance with the performance indicator^51–56^. According to the parameter values presented in Table 1, a population of spiders, each of which demonstrates an ideal solution, is employed as a starting point for the proposed ABWO. This is the first step in the process, and it consists of the spiders mating and seeking to make a new breed of offspring. The purpose of this new generation is to bring the population up to date, and it will also choose two genes at random in compliance with the cannibalism and mutation plan that has been established. Once again, the population is altered in line with the values of the parameters, and the procedure is carried out as frequently as is required until the stopping criterion is attained (all constraints are satisfied and global optimal point is determined).
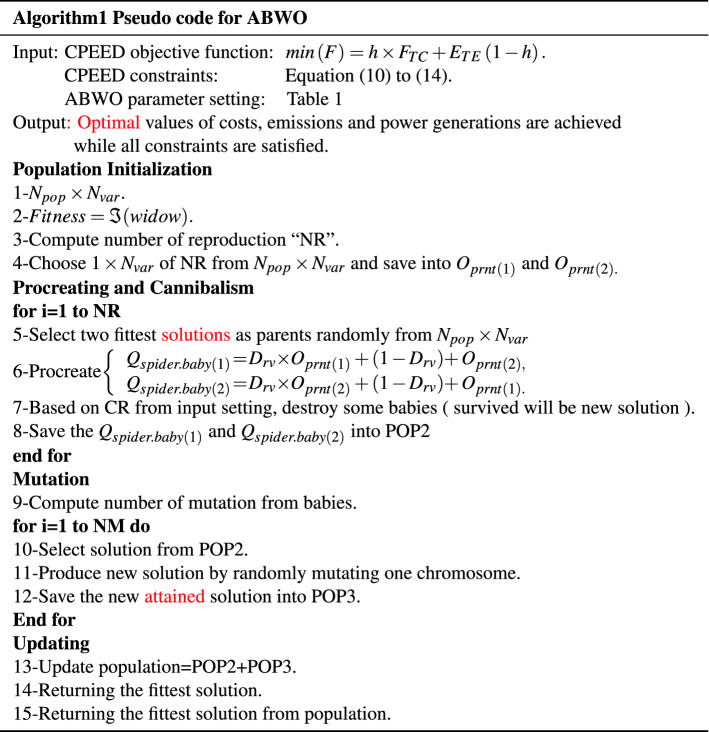


It is worth mentioning that the present work considers a multi-objective model for optimization by considering a large number of constraints, compared to the existing works in^46–49^. The optimization problem considers both the conventional and renewable power plants, containing both solar and wind sources, for an energy hub. In addition, system level constraints, namely, capacity constraint, ramp-rate limitation, load balancing limitation, VLE effect of thermal plants and RES uncertainties, have also been incorporated in our study. Furthermore, the optimization problem considers both the cost and the greenhouse gases emission optimization for dealing with the environmental challenges. To demonstrate the effectiveness of the suggested ABWO approach in the present work, five small- to large-scale case studies have been examined in the next section. In terms of optimal operational cost, faster convergence, and less computational time, the analysis demonstrated that ABWO performs significantly better than the existing advanced approaches, reported in the literature^51–56^. Consequently, the consideration of several constraints, different type of systems, reduction of generation cost, and minimization emission of greenhouse gases along with improvement in results in terms of optimal cost, less convergence time, and less computational resources is a significant contribution of the present work.

## Simulation and results

**Figure 4:**
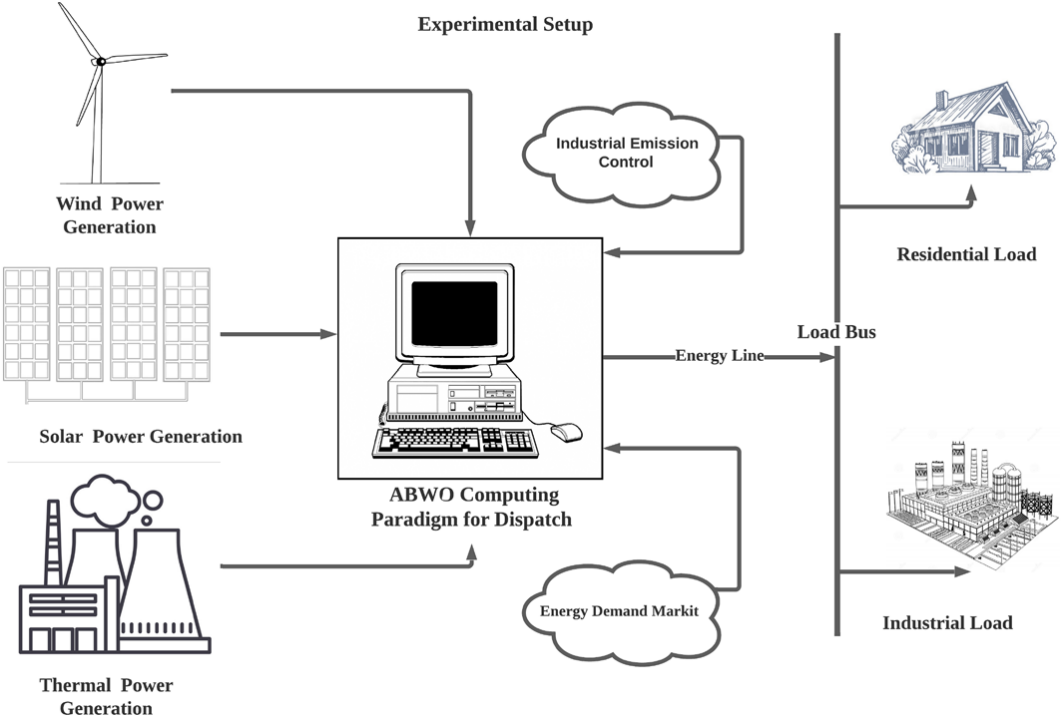
Generic experimental layout.

Five benchmark test systems have been chosen to demonstrate the practicability and efficacy of ABWO in real-world applications. The obtained results from ABWO are compared to those obtained from existing state-of-the-art meta-heuristic techniques. The required time analysis was conducted on Lenovo notebook outfitted with an Intel celeron (R) N2940 CPU running at 1.83 GHz and 4.0 GB of RAM, with MATLAB version R2017b. Five categories of experimental case studies with experimental layout shown in Fig. 4 are adopted for this research. In addition to solar and wind generators, five case studies are comprised of a varying number of traditional thermal generators. In order to evaluate the speed, agility and efficacy of the proposed algorithm, these generating units with a wide range of operating conditions, as shown in the constraints, are chosen for each test system. The ABWO algorithm is applied to both small- and large-scale benchmark systems in order to ascertain whether its controls are adequate or not for all systems integration under consideration. In order to verify the accuracy of the proposed method, the case studies at varying conditions with different numbers of generators are chosen with the primary goal of establishing validity of the proposed method. The precise details of these five case studies are discussed in detail in subsections as follow.

### Case study-I

**Figure 5:**
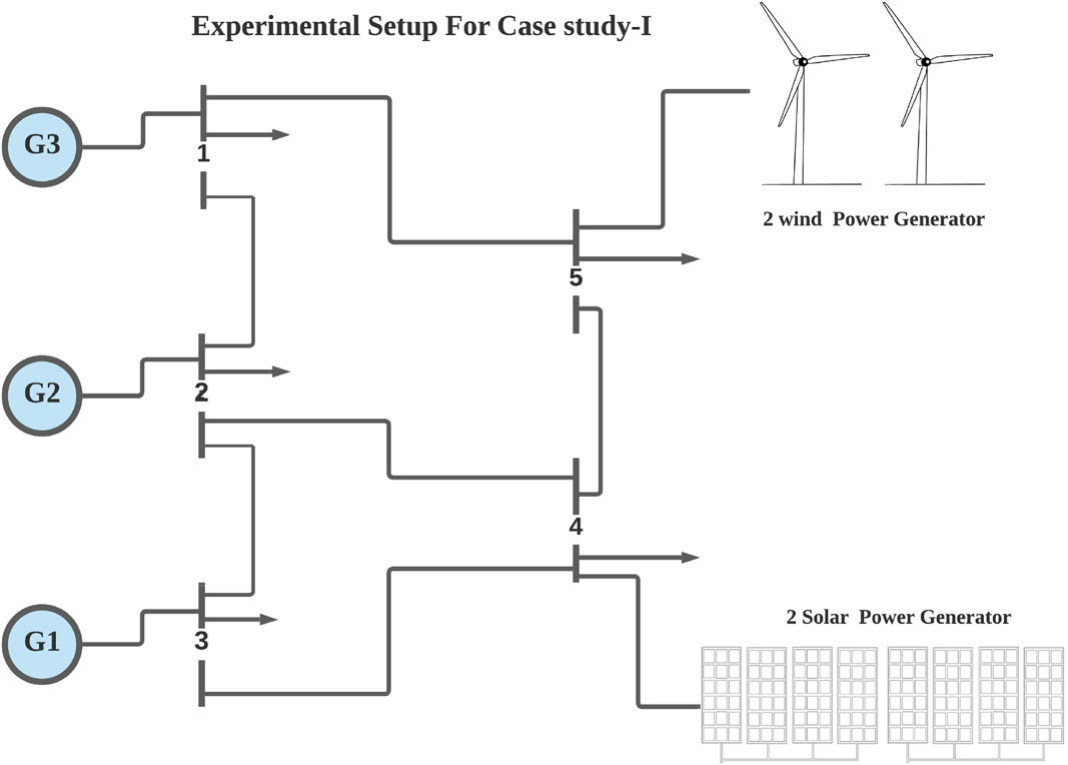
Experimental layout of case study-I.

**Figure 6:**
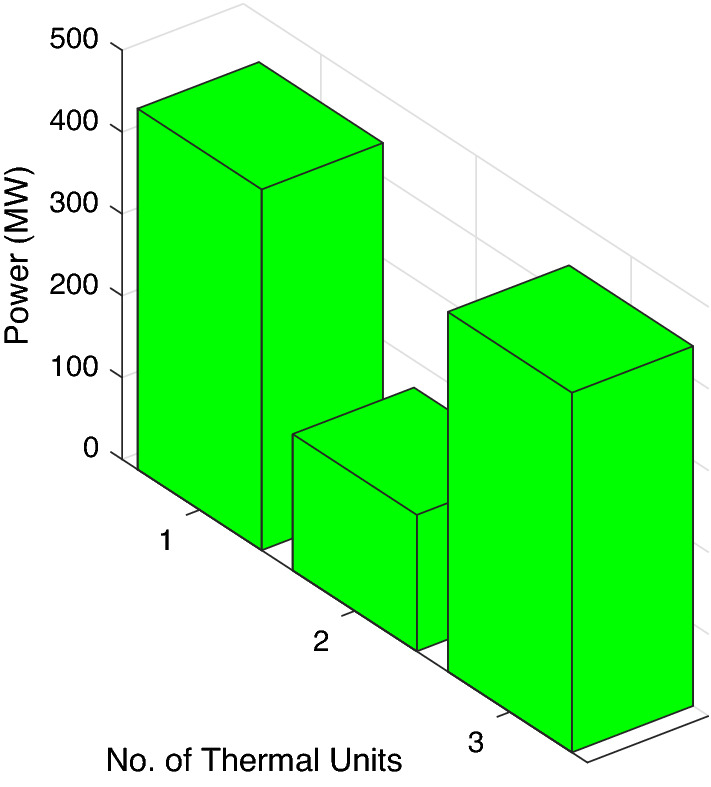
Optimum power generations for case study-I.

**Figure 7:**
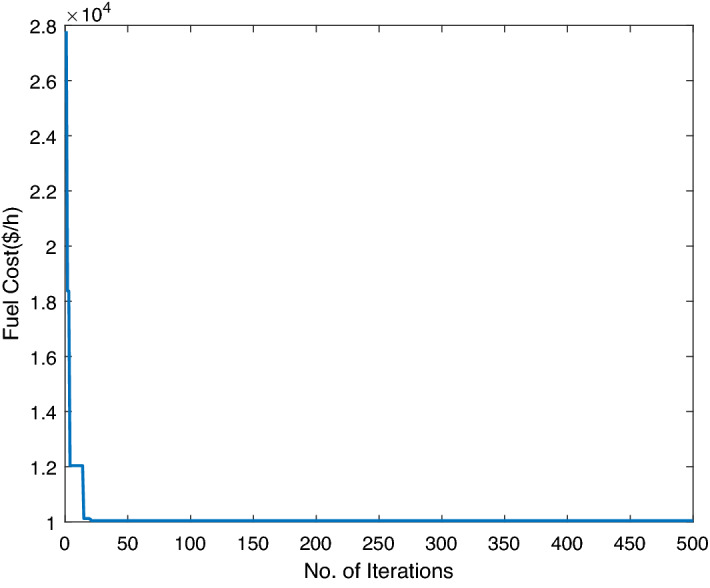
ABWO performance for case study-I.

In this case study, three traditional thermal power plants are integrated with two solar and two wind RES. Energy flow constraints are neglected, but energy capacity, valve-point effect, forbidden operating regions, and machine ramp-rate limits are all considered. Tables 2 and 3 contain information on the input data for wind and PV units, respectively, while Table 4 contains information on the cost coefficients for thermal units. Each wind unit has a generating capacity of 0.8 megawatts (MW). The experimental layout for Case study-I is shown in Fig. 5. Each solar power unit has a maximum capacity of 10 MW. The bid rate for a solar power unit is 2.854 dollars per megawatt hour (MWh). The optimum power generation outputs of thermal units are shown in Fig. 6. The total demand for electricity is 1050 MW, and the optimum power output obtained by ABWO approach is shown in Fig. 6. Table 5 provides the comparison of best mean costs (which is 10017.4602 by ABWO) by satisfying all non-convex constraints with other advanced heuristic approaches. The 500 independent iterations have been performed to testify efficacy of the proposed ABWO approach. The convergence profile of the proposed ABWO computing paradigm is shown in Fig. 7. It is quite evident from Fig. 7 that the suggested computing paradigm handles the complex objective function bounded by nonlinear limitation within fine computational time as compared to other heuristic techniques in Table 5.

**Table 2:** Wind power units input data.

Units	$V_{in}$	$V_{out}$	$V_{r}$	$\Pi$	$\mathrm T$	$w_r$	$C_a$
1	5	45	15	1.5	15	10	1.25
2	5	45	15	1.5	15	10	1

**Table 3:** Solar power input data.

Unit	*R*	*TE*	$\chi$	$N_{series}$	$N_{Parallel}$
1	6.30	3.43	0.043	20	20
2	5.38	5.43	0.043	20	20

Unit	$U_{normal.temp}$	$U_{ambient}$	$U_{cell.stcon}$	$S_{rad.stcon}$	${{S_{p(stcon)}}}$
1	45.5	20	25	1000	10
2	45.5	20	25	1000	10

**Table 4:** Thermal units cost input data for case study-I.

Unit	$u_i$	$v_i$	$w_i$	$e_i$	$f_i$	$p_i^{min }$	$p_i^{max }$
1	0.0016	7.92	561	300	0.032	100	600
2	0.0048	7.92	78	150	0.063	150	200
3	0.0019	7.85	310	200	0.042	100	400

**Table 5:** Performance analysis of ABWO comparing other approaches for case study-I.

Methods	Generation cost ($/h) mean cost	Computational time (s)
**ABWO**	**10,017.4602**	**7.43**
DA^51^	10,049.1948	8
CSA^52^	10,065.7013	10
ALO^53^	10,079.2323	12
ORCCRO^57^	10,080.8028	14.6
BBO^56^	10,088.8563	17.8
PSO^54^	10,089.0073	20.5
GA^55^	10,096.199	24.2

Significant values are in bold.

**Figure 8:**
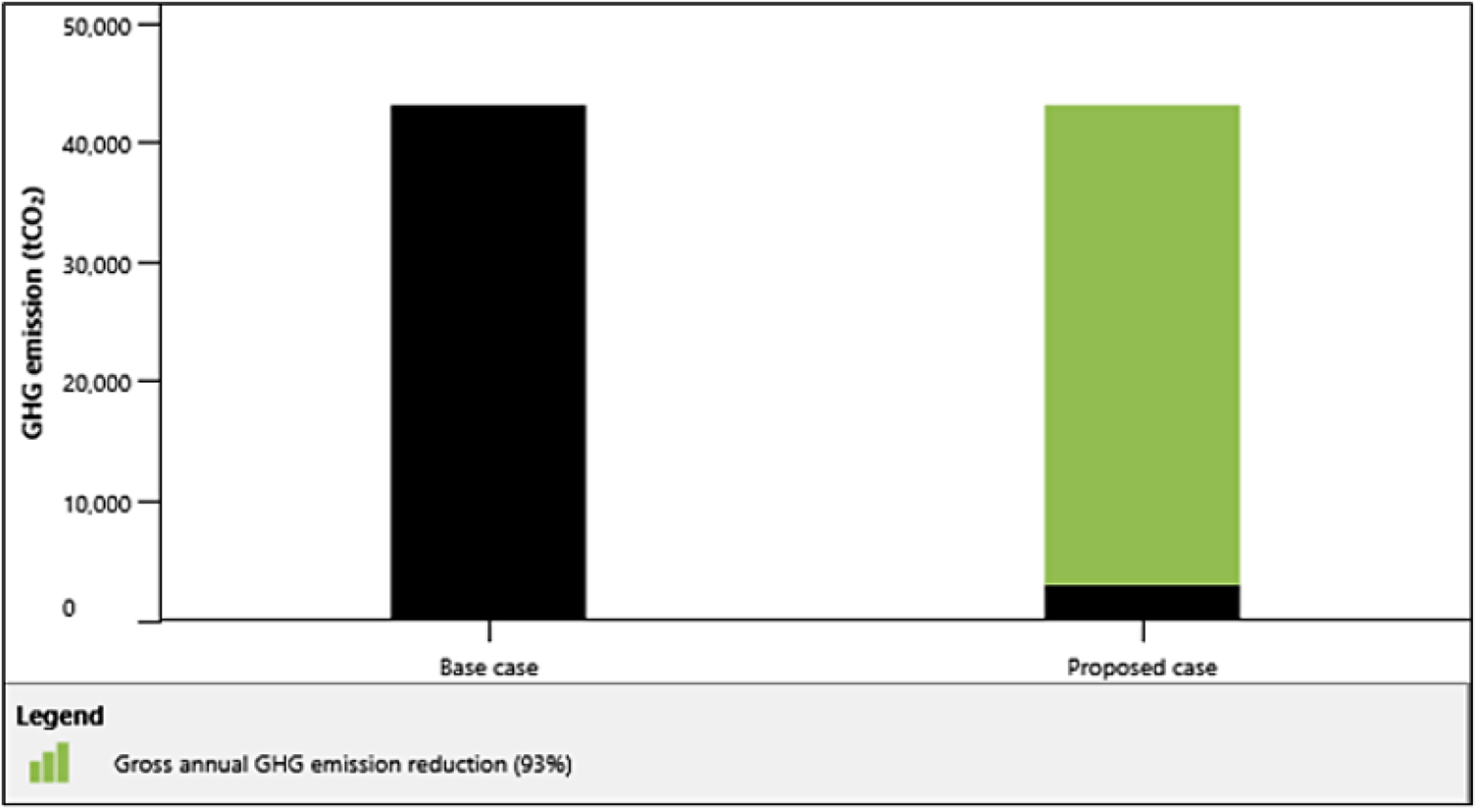
emissions comparison of thermo-electric plant via RETScreen®.

To compare the greenhouse gases emission of plants with the same capacity with RES, we have simulated the same power capacity hub for RES using thermal power plant as the base case via RETScreen®^72^ (one of the most reliable tool developed by Canadian government for green energy design evaluation) and determined that the gross annual reduction in carbon is 40,180.5 t$CO_2$, which is nearly 93% of thermal plant. Figure 8 depicts the emissions comparison of base case (thermo-electric plant) and RES generation.

### Case study-II

A combination of five traditional thermal power generating units, two wind power generators and two solar PV setups is used in this case study. The non-convex constraints of VLE, capacity limits, machine ramp limits and forbidden operating region are considered to show the adaptability and efficacy of the proposed scheme. The maximum capacity of solar power units is 10 MW with bid rate of 2.854 dollars per megawatt hour. The load demand for utility network is 730 MW. The main purpose of selecting four variable-condition test sets with various generators is to assess the convergence of ABWO over a finite amount of time. The input data for wind and photovoltaic units has been presented in Tables 2 and 3, respectively. While the cost coefficients data for thermal units is presented in Table 6.

**Figure 9:**
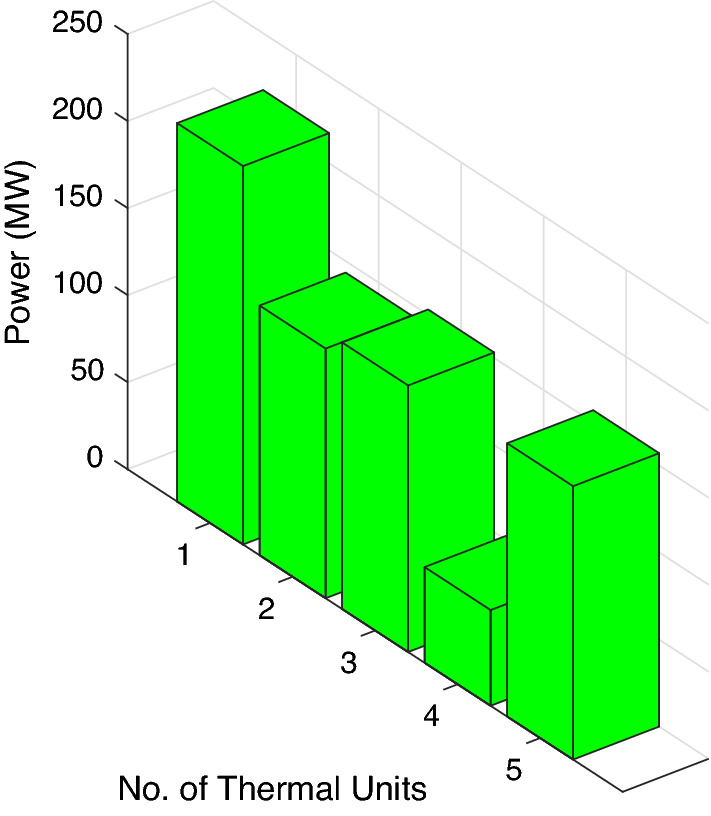
Optimum power generations for case study-II.

**Figure 10:**
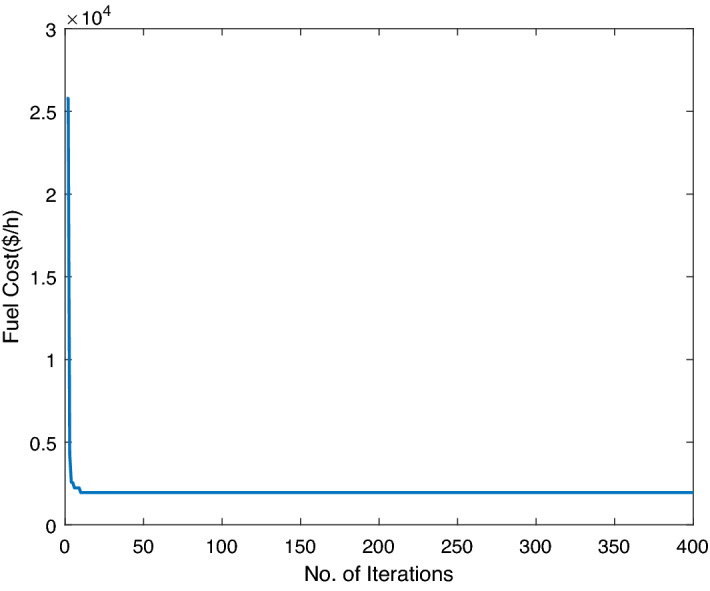
ABWO performance for case study-II.

Table 7 depicts the best mean value 1944.61 attained by ABWO for case study-II. It clearly shows that the performance of ABWO is again better as compared to other computational techniques in literature. The computational efficacy of ABWO outperforms well while handling complex system limitations of thermal power plants. Figure 9 represents the optimal combination of powers attained for case study-II, and the power generated from RES in this case study is 4.3621MW. Figure 10 depicts the convergence profile of the proposed computing framework. Here, it is evident that the proposed technique rapidly converges to the optimum solution.

**Table 6:** Thermal units cost input data for case study-II.

Unit	$u_i$	$v_i$	$w_i$	$e_i$	$f_i$	$p_{i}^{\min }$	${p_{i}^{\max }}$
1	0.0015	1.8	40	200	0.035	50	300
2	0.0030	1.8	60	140	0.040	20	125
3	0.0012	2.1	100	160	0.038	30	175
4	0.0080	2	25	100	0.042	10	75
5	0.0010	2	120	180	0.037	40	250

### Case study-III

**Figure 11:**
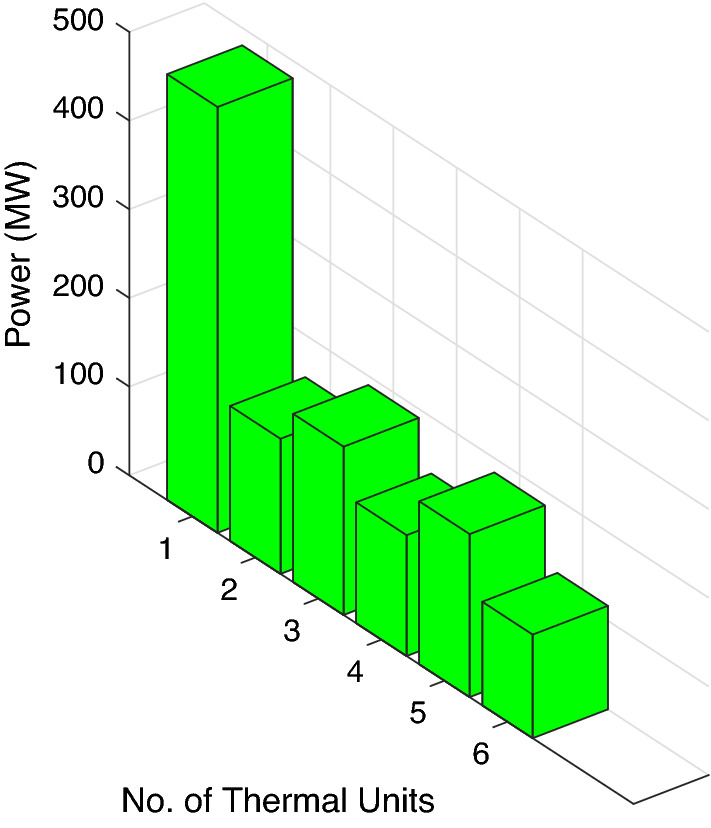
Optimum power generations for case study-III.

**Figure 12:**
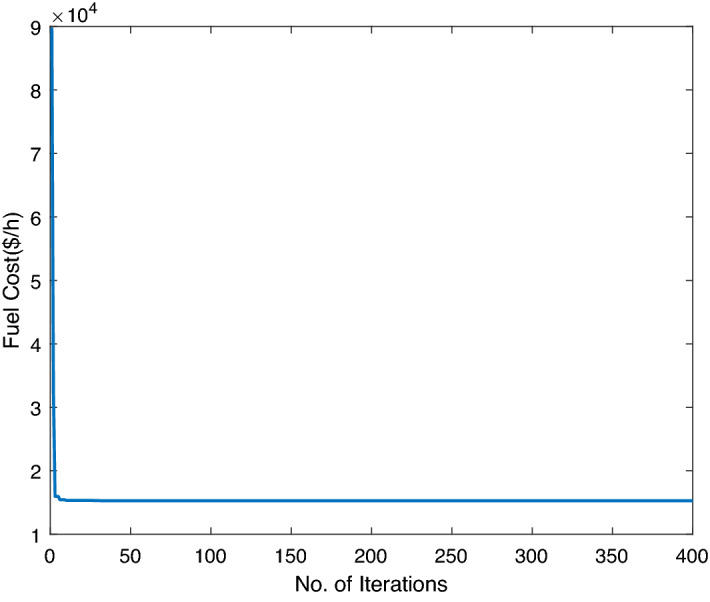
ABWO performance for case study-III.

Here, six conventional thermal units are combined with two wind and two solar units by accounting all linear and non-convex constraints. A maximum of 0.8 megawatts of energy can be extracted from a wind turbine. Each solar photo-voltaic (PV) system generates 10 MW. The bid rate for the solar power unit has been taken as 2.854 dollar per megawatt. The RES data is tabulated in Tables 2 and 3. The utility network’s load demand is 1263 MW. Table 8 contains the fuel coefficient input data for thermal units. Table 9 shows the performance of ABWO with respect to other heuristic approaches. Again, the proposed computing framework performs better than the conventional computing techniques in terms of computational and fuel costs, while efficiently handling the non-convex limitations of the objective function. Figure 11 depicts the best allocated power outputs for case study-III, and Fig. 12 shows the convergence profile against non-convex objective function in Eq. (6).

**Table 7:** Performance analysis of ABWO comparing other approaches case study-II.

Methods	Generation cost ($/h) mean cost	Computational time (s)
**ABWO**	**1944.6161**	**7.84**
DA^51^	2018.0762	12
CSA^52^	2021.5229	12.6
ALO^53^	2025.8236	14.5
ORCCRO^57^	2046.6344	17.2
BBO^56^	2058.5299	21
PSO^54^	2060.80	25
GA^55^	2073.8957	28

Significant values are in bold.

**Table 8:** Thermal units cost input data for case study-III.

Unit	$u_i$	$v_i$	$w_i$	$e_i$	$f_i$	${p_i^{\min }}$	${p_i^{\max }}$
1	0.0070	7.0	240	300	0.031	100	500
2	0.0095	10.0	200	200	0.042	50	200
3	0.0090	8.5	220	150	0.063	80	300
4	0.0090	11	200	150	0.063	50	150
5	0.0080	10.5	220	150	0.063	50	200
6	0.0075	12	190	150	0.063	50	120

**Table 9:** Performance analysis of ABWO comparing other approaches case study-III.

Methods	Generation cost ($/h) mean cost	Computational time (s)
**ABWO**	**15,236.2203**	**9.0028**
DA^51^	15,268.8325	15
CSA^52^	15,277.2396	18
ALO^53^	15,278.2188	19.2
ORCCRO^57^	15,280.1136	23.9
BBO^56^	15,284.9752	24
PSO^54^	15,286.3639	27.6
GA^55^	15,287.8373	28

Significant values are in bold.

### Case study-IV

**Figure 13:**
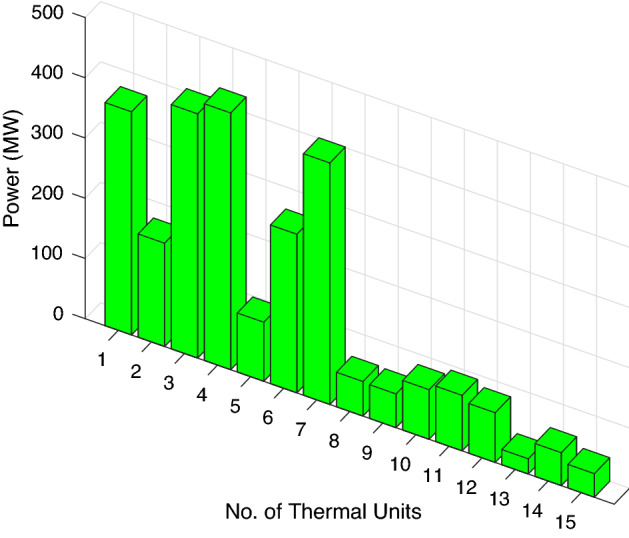
Optimum power generations for case study-IV.

**Figure 14:**
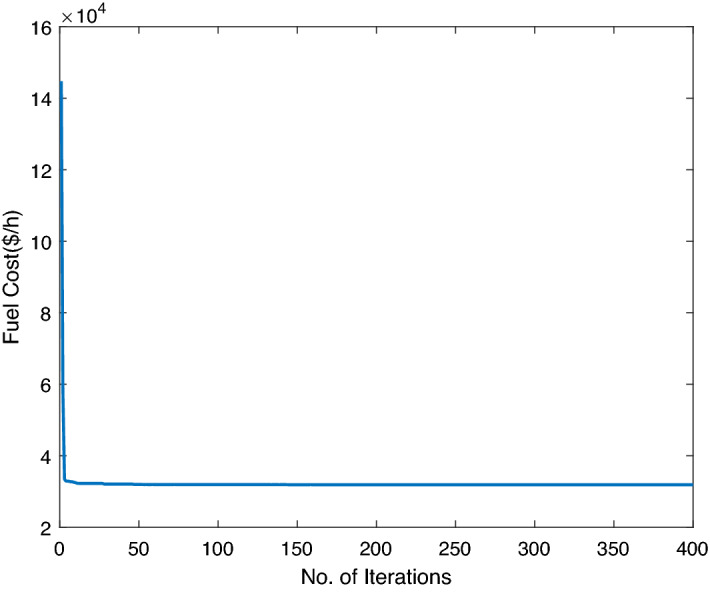
ABWO performance for case study-IV.

In this case study, a medium-scale test system of fifteen traditional thermal power plants has been considered along with two solar and wind RES. The parameters of bid rate for solar and quantity of RES are same as in the previous case studies. The input data for RES is provided in Tables 2 and 3, while the thermal unit fuel input data is tabulated in Table 10. Load demand power for this case study is accounted as 2630 MW. Table 11 shows that the best mean cost attained by ABWO is much finer as compare with the traditional heuristic techniques. Another important factor for soft computing framework is the computational budget. It can also be verified from Table 11 that the computational time required for Case study-IV is much better than the heuristic approaches. Figure 13 depicts the optimum power allocation by ABWO, and Fig. 14 represents the convergence profile for Case Study IV.

**Table 10:** Thermal units cost input data for case study-IV.

Unit	$u_i$	$v_i$	$w_i$	$e_i$	$f_i$	${p_i^{\min }}$	${p_i^{\max }}$
1	0.000299	10.1	671	100	0.084	150	455
2	0.000183	10.2	574	100	0.084	150	455
3	0.001126	8.8	374	100	0.084	20	130
4	0.001126	8.8	374	150	0.063	20	130
5	0.000205	10.4	461	120	0.074	150	470
6	0.000301	10.1	630	100	0.084	135	460
7	0.000364	9.8	548	200	0.042	135	465
8	0.000338	11.2	227	200	0.042	60	300
9	0.000807	11.2	173	200	0.042	25	160
10	0.001203	10.7	175	200	0.042	25	162
11	0.003586	10.2	186	200	0.042	20	80
12	0.005513	9.9	230	200	0.042	20	80
13	0.000371	13.1	225	300	0.035	25	85
14	0.001929	12.1	309	300	0.035	15	55
15	0.004447	12.4	323	300	0.035	15	55

**Table 11:** Performance analysis of ABWO comparing other approaches case study-IV.

Methods	Generation cost ($/h) mean cost	Computational time (s)
**ABWO**	**31846.7933**	**16.48685**
DA^51^	32,310.2922	20
CSA^52^	32,318.1056	24.3
ALO^53^	32,329.5536	26.8
ORCCRO^57^	32,358.0926	29
BBO^56^	32,260.2696	31.4
PSO^54^	32,365.9862	35
GA^55^	32,388.5473	36.7

Significant values are in bold.

### Case study-V

This case study has been considered with the assumption that the generation from RES is zero because of atmospheric conditions (or system is disconnected from power grid for maintenance purposes). The conventional thermal plants will share all load of network area with restrictions of emissions imposed by emissions regulatory authorities such as KOYOTO PROTOCOL^73^, EPA^74^ and European Commission^75^ for sustainable environment. A gas fueled plant is selected with non-convex and linear constraints. The emissions and fuel coefficient data is tabulated in Table 12 with load demand 850MW. The two highly conflicting objective functions in Eq. (8) are chosen. The ABWO outperforms to attain the optimum fuel cost and emissions while handling constraints presented in Eqs. (9) to (16). The ABWO framework is used to simultaneously minimize the total cost of fuel and emissions. Table 13 outlines the fuel costs and minimum emissions obtained by the proposed approach of ABWO, NSGA-II^76^ and TABU^77^, for this case study. The suggested approach can be found from Table 13, which is less expensive than other comparative approaches with respect to fuel and emissions. The fuel minimization problem obtained values are 8241.0821 and 0.0953 lb/h, respectively, for the minimum fuel cost and emissions.

**Table 12:** Thermal units cost and emissions data for case study-V.

Unit	$u_i$	$v_i$	$w_i$	$e_i$	$f_i$
1	561	7.92	0.001	300	0.031
2	310	7.85	0.001	200	0.042
3	78	7.97	0.004	150	0.063

Unit	${p_i^{\min }}$	${p_i^{\max }}$	$\alpha _i$	$\beta _i$	$\gamma _i$
1	100	600	0.043	− 9.49e−5	1.47e−7
2	100	400	0.055	− 9.73e−5	3.02e−7
3	50	200	0.027	− 3.53e−4	1.93e−6

**Table 13:** ABWO comparison with other heuristic techniques for case study-V.

Unit	TABU search^77^	NSGA-II^76^	ABWO
$P_1$	435.69	435.885	435.39
$P_2$	298.828	299.98	265.94
$P_3$	131.28	129.95	148.70
Total generation	850 MW	850 MW	850 MW
Best cost	8344.59	8363.62	**8255.10**
Best emissions	0.0986	0.0959	**0.0953**

Significant values are in bold.

**Table 14:** Statistical analysis of all case studies.

Cost (max)	STD	Time
**Case study-I**		
2.7007e+04	0.941	7.4
**Case study-II**		
2.5307e+04	1.756	7.84
**Case study-III**		
9.0166e+04	14.324	9.000
**Case study-IV**		
1.4475e+05	10.21	16.4
**Case study-V**		
1.5223e+04	3.45	5.97
–	–	–
**Emissions (max)**	**STD**	**Time**
1.005	–	5.97

**Figure 15:**
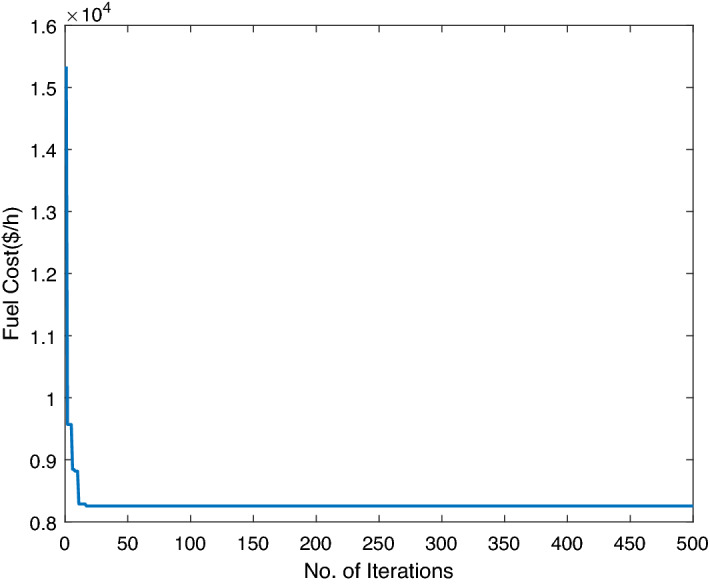
ABWO performance for case study-V.

**Table 15:** RES contribution in all case studies.

**Case study-I**	
RES generation	3.9537 MW
Load demand	1050 MW
**Case study-II**	
RES generation	4.362 MW
Load demand	730 MW
**Case study-III**	
RES generation	3.664 MW
Load demand	1263 MW
**Case study-IV**	
RES generation	4.544 MW
Load demand	2630 MW
**Case study-V**	
RES generation	0
Load demand	850 MW

Figure 15 depicts the convergence behaviour of cost and pollutant emissions. The approach of ABWO in Table 13 is preferable, compared to the other approaches. This is due to the true modelling of the black widow capacities to determine the best solution. ABWO looks for newly updated solutions around the region, which allows enough exploitation.

### Statistical analysis

Statistical analyses are the process by which trends, patterns and relations are determined through use of statistical data. Such analyses constitute a crucial instrument of data analysis, used by scientists on a limited objective basis. A comprehensive statistical analysis is used to assess the reliability of stochastic algorithms. Table 14 shows the trends and patterns for all case studies investigated for this work. Table 15 depicts the contribution of RES in power grid by sharing load of thermal power plants, hence, reducing the carbon and other pollutant oxides. The RES contribution against different loads is provided for each case study.

The proposed scheme has various advantages as compared to other optimization frameworks. Some of elegant features as compared to the conventional approaches are early convergence and the ability to achieve an optimal cost and emissions, which saves an energy hub’s annual revenue. Additionally, the provided scheme can achieve optimal solution in the fewest iterations subject to different restrict system limitations. With a solid balance between exploitation and exploration, the framework is less likely to get stuck in local minima compared to other advance evolutionary-based algorithms.

## Conclusions

The purpose of this study was to consider an existing constrained multi-objective population based optimization technique known as the ABWO computing paradigm for finding the optimal solutions to multi-objective CPEED problems with RES penetration by minimizing two conflicting total cost and environmental emissions objectives. In addition, the feasibility and reliability of the proposed techniques were verified for five test cases, including small-, medium- and large-scales systems. The research outcomes contributed to the improvement of the power system for attaining environmental and economic benefits by introducing an advanced concept for low carbon generation by formulating CPEED with RES. The ABWO computing algorithm was evaluated using uni-model and multi-model benchmark functions, and it has been shown to outperform the existing algorithms in terms of convergence speed and computational budget. By utilizing RES for electricity generation, the cost of fuel and toxic air emissions were significantly reduced. RES penetration in traditional fuel-based plants not only helped in protecting the earth atmosphere but also reduced the dependency on imported fossil fuels which further aids to handle geopolitical problems. ABWO can assist the power generation companies to control emissions imposed by international environmental protection agencies operating globally. The adoption of renewable energy will also aid in the development of the economy and creation of jobs in green energy manufacturing and installation. Despite the fact that the ABWO achieves satisfactory dispatch results, there are some limitations to this study. As a starting point, it is essential to further enhance the precision and integration security of the ABWO optimization technique by incorporating device-level limitations of plants. ABWO has limited flexibility to these constraints, and it is difficult to simulate them. As these features will strengthen the grids reliability and transparency, and they also make it easier to deal with the cyber threats. Second, the CPEED problem does not account sufficiently for the effects of energy storage system, that is, probabilistic loads of plug-in electric automobiles, and it is complex to model driver behavior and storage system constraints in current framework. The implementation of ABWO algorithms for complex problems under various parameters, like population size, crossover rate, mutation rate, and cannibalism rate, requires parametric tuning procedures. Identification of viable solutions for these parameters is currently a challenging research issue for a scientific problem. In the future, we will also use RETScreen to assess the price of RES with storage for grid operation, such as electric mobility vehicles. Future studies will also combine the four-season climate database with different wind speeds or solar irradiance to precisely model the uncertainty of renewable sources.

## Data Availability

The data-sets used and/or analysed during the current study is available from the corresponding author on reasonable request. All of data-set used in the study has been either provided or cited in the article.
